# Evaluation of dialectical behavior therapy for adolescents in routine clinical practice: a pre-post study

**DOI:** 10.1186/s12888-024-05876-z

**Published:** 2024-06-14

**Authors:** Anne Mari Syversen, Viktor Schønning, Gro Sydnes Fjellheim, Irene Elgen, Gro Janne Wergeland

**Affiliations:** 1 Department of Child and Adolescent Psychiatry, Division of Psychiatry, Haukeland University Hospital, N-5021 Bergen, Norway; 2 Regional Centre for Child and Youth Mental Health and Child Welfare, Norwegian Research Center, NORCE, Bergen, Norway; 3 Department of Clinical Medicine, Faculty of Medicine, University of Bergen, Bergen, Norway

**Keywords:** DBT-A, Emotional dysregulation, Self-harm, Suicidal ideation, Adolescents, Routine clinical practice

## Abstract

**Background:**

Self-harm and suicidal ideation are prevalent among adolescents, cause physical and psychosocial disability, and have potentially life-threatening consequences. Dialectical behavioral therapy for Adolescents (DBT-A) is an evidence-based intervention for reducing self-harm. However, few studies have investigated the effectiveness of DBT-A when delivered in routine clinical practice.

**Methods:**

A follow-up cohort study, based on data from a quality assessment register of DBT-A in child and adolescent mental health services including seven outpatient clinics. Inclusion criteria were ongoing or a history of self-harming behavior the last 6 months; current suicidal behavior; at least 3 criteria of DSM-IV Borderline personality disorder (BPD), or at least the self-destruction criterion of DSM-IV BPD, in addition to minimum 2 subthreshold criteria; and fluency in Norwegian. Participants received 20 weeks of DBT-A consisting of multifamily skills training groups and individual therapy sessions. Outcomes from 41 participants included frequency of self-harm, suicide attempts and hospitalizations caused by self-harm or suicide attempts, assessed pre-, during, and post-treatment by self-report and reviews of the patient’s medical records. Suicidal ideation, urge to self-harm and perceived feelings of happiness and sadness were assessed by the patients’ diary cards at week 1, 5, 10, 15 and 20 of the treatment program.

**Results:**

Participants attended an average of 17.9 (*SD* = 4.7) individual sessions, 14.7 (*SD* = 3.4) group-based skills training sessions and 4.6 (*SD* = 4.1) brief intersession telephone consultations. Moderate to large within-group effect sizes (ES) were found in self-harm from pre-treatment to 1–5 weeks (*d* = 0.64), 6–10 weeks (*d* = 0.84), 11–15 weeks (*d* = 0.99), 16–20 weeks (*d* = 1.26) and post-treatment (*d* = 1.68). Nine participants were admitted to hospitalization during DBT-A, whereas five had attempted suicide, but no suicides were completed. No statistically significant changes were found in suicidal ideation, urge to self-harm or perceived feelings of happiness or sadness from pre to post treatment.

**Conclusion:**

The findings of the current study are promising as the participants reported considerably reduced self-harm behavior after DBT-A treatment in a child and adolescent mental health outpatient setting.

**Supplementary Information:**

The online version contains supplementary material available at 10.1186/s12888-024-05876-z.

## Background

Self-harm, suicidal ideation, and suicide attempts among adolescents are serious public health concerns, as suicide is one of the leading causes of deaths among adolescents [1]. Self-harming behavior (i.e., nonfatal self-injury with or without suicidal intent) in adolescence is associated with mental health problems, such as depression, anxiety, substance abuse, and antisocial behavior in addition to an increased risk of poorer educational and occupational outcomes [2, 3]. Repeated self-harm is further associated with Borderline personality disorder (BPD) [4], as emotional instability has shown to maintain self-harming behavior and an increased risk of suicidal behavior in individuals with BPD [5].

A meta-analysis including 144 population-based studies from 41 countries reported a lifetime prevalence of 16.9% for self-harm in adolescents aged 12 to 18 years old [6]. A recent study reported an increase in self-harm among Norwegian adolescents from 4.1% to 16.2% from 2002 to 2018 [7]. Given the prevalence, impairment, and consequences of adolescent self-harming behavior, offering developmentally appropriate and effective treatment is critical.

Specialized treatments for adolescents targeting self-harm behavior exist with Dialectical behavioral therapy (DBT) and Mentalization-based therapy (MBT) as the most promising treatments [8, 9]. A systematic review from 2022 that included 21 studies on DBT for adolescents and 4 studies on MBT found significant improvements in suicidal ideation, suicidal attempts, and self-harm in both treatments [10]. However, a recent systematic review and meta-analysis on the efficacy of MBT in treating self-harm showed no superiority of MBT to control conditions (e.g., treatment as usual, structured clinical management) [11]. Dialectical Behavior Therapy for Adolescents (DBT-A) is the treatment that has shown the most encouraging results [12, 13], and is a well-established [14] and recommended treatment for reducing self-harming behavior and BPD according to several guidelines [15–17].

DBT for suicidal and self-harming behaviors, was developed for adults with BPD [18], but has since been adapted for adolescents aged 12 to 19 years (DBT-A; [19]). DBT-A is a manualized 16 to 20-week behavioral treatment program, comprising skills training in groups, individual therapy sessions, and telephone consultations between sessions. The treatment is focusing on skills for emotion regulation and dysfunctional behavior, and support for generalizing the skills [8]. The treatment goal is to replace dysfunctional behavior with more adaptive behavior [20].

A recent systematic review and meta-analysis including 21 studies conducted in inpatient and outpatient settings, and university research clinics and routine clinical care with in total 1673 adolescents receiving DBT-A, found small to moderate between group effects for reducing self-harm frequency compared to control conditions, and large within-group effects of DBT-A in pre-post evaluations [12]. The meta-analysis included five randomized-controlled trials (RCTs), three controlled clinical trials, and 13 pre-post studies [12]. Across the different study designs the results showed promising effects of DBT-A for self-harm and suicidal ideation. It is encouraging that an increasing number of studies are being carried out to evaluate DBT-A and examine its clinical effectiveness, i.e., how DBT-A performs when delivered in routine clinical care. However, although data on the effectiveness of DBT-A are emerging the research is still limited and more knowledge on how DBT-A performs when delivered in routine clinical care is called for [12, 13].

A well-established treatment such as DBT-A may perform differently in routine clinical care compared to delivery in research clinics for a variety of reasons [21, 22]. For example, studies conducted to establish efficacy are often carried out with a methodologically stringent procedure to ensure high internal validity, e.g., by using rigorous inclusion and exclusion criteria, randomizing participants into different conditions, and having therapists with access to extensive training, supervision, and treatment monitoring with an emphasis on treatment integrity [23, 24]. This methodological rigor of efficacy trials to maximize experimental control may differ from “real world” clinical settings. As such, treatment programs developed and evaluated under highly controlled conditions in specialized research settings may not produce similar results when delivered in routine clinical care [21]. Thus, it is important to study the effectiveness of DBT-A, at sites beyond those where evidence was derived. Studies evaluating DBT-A with treatment implemented as part of the routine clinical services delivered could complement findings from efficacy studies regarding the effectiveness of the treatment [25, 26].

The aim of this study was to examine self-harm, suicide attempts and hospitalizations from suicidal behavior before-, during- and after a 20 week-duration outpatient DBT-A program for adolescents with suicidal and self-harming behavior. Secondary aims were to examine whether there would changes in suicidal ideation, self-harm urges, self-perceived sadness, and happiness from pre-, to post-treatment.

## Methods

### Setting and design

This follow-up cohort study was based on data from a quality-assessment register that was conducted at the DBT-A team at the Department for Child and Adolescent Mental Health Services (CAMHS) at Haukeland University Hospital, Norway. Patients are referred to the DBT-A team from the seven child and adolescent psychiatric outpatient clinics in the hospital’s catchment area, covering both urban and rural areas. Adolescents are mainly referred to these clinics by general practitioners or less often by child welfare services. Services are free of charge for all families and there is marginal use of private mental health care for children in Norway. The seven clinics serve a population of 96,544 youth below 18 years [27], and receive an average of 5899 unique patients each year (2022).

The quality assessment register was established in 1st of January 2017 for ongoing evaluation of the DBT-A services.

### Procedure

Patients referred to DBT-A had gone through a diagnostic assessment and conclusion at the outpatient clinics prior to referral to DBT-A. If the patients met the inclusion criteria for self-harm and/or suicidal behavior and consented to referral to the DBT-A team, they were referred and considered for treatment. Patients typically attended 3–4 pre-treatment sessions with a DBT-A therapist where they were screened for eligibility and established a commitment to participate in the full 20 weeks of the DBT-A treatment. In order to establish commitment to the treatment, the therapists followed the DBT-A manual’s commitment strategies, e.g., work on the pros and cons regarding the treatment, playing the devil’s advocate, and highlighting the freedom to choose and absence of alternatives [4]. In addition to wanting treatment, full commitment involves completing homework exercises, filling out DBT-A diary cards weekly, using a safety plan during crises, handling in knives, razors and other self-harm tools to the therapist, being willing to discuss and analyze their self-destructive behavior, and attempting alternative solutions and practice skills.

Patients that were accepted for DBT-A treatment had their outpatient treatment paused as there should be no parallel psychological treatment during DBT-A. Patients in need of additional pharmacotherapy were allowed to continue their administration by their referring clinic.

*Inclusion criteria* for DBT-A were ongoing or a history of self-harming behavior the last 6 months; current suicidal behavior (suicidal thoughts or at least one suicide attempt within the last 6 months); at least 3 criteria of DSM-IV BPD, or at least the self-destruction criterion of DSM-IV BPD, in addition to minimum 2 subthreshold criteria; and fluency in Norwegian. *Exclusion criteria* were intellectual disability, significant learning impairments, significant language impairments, autism, a psychotic disorder, or substance abuse disorder. Anorexia nervosa was not an exclusion criterion but could not be the primary diagnosis.

Data was collected from the register in 2017 until the 1st of July 2023, with a total of 103 patients who accepted and started DBT-A. Consent to participate in the current study was provided retrospectively during December 2022 and January 2023. Eligible participants (i.e., those that had received DBT-A) received an SMS with information of the study, including participation implied use of data from their treatment, an invitation to participate, and a link to the consent form. The consent forms were digital. For adolescents under 16 years of age, both parents provided consent to participation as per Norwegian law.

### Treatment

DBT-A [8] was delivered for 20 weeks and consisted of one weekly session of individual therapy (45 min), one weekly session of multifamily skills training groups with a caregiver (120 min) and telephone coaching with individual therapists outside therapy sessions as needed. The patients’ complete weekly diary cards as part of DBT-A. The treatment was delivered according to the DBT-A manual by Miller [8], with the exception of the telephone coaching that according to the manual should be offered on a 24 h basis, in the current study was offered workdays until 8 pm. The treatment was part of the public health care services and free of charge to the patients and their families.

The treatment was provided by three psychologists and two psychiatric nurses that were certified as DBT-therapists through an 80 h seminar with additional 12 months of supervised practice on clinical training cases by The National Centre for Suicide Research and Prevention in Oslo, Norway. The therapists had a minimum of 2 years of experience as DBT-therapists prior to the establishment of the quality assessment register and the current study. The DBT team received supervision from a senior psychologist and DBT-therapist during the first two years of the data collection. The therapists attended weekly DBT consultation team meetings to enhance fidelity to the treatment and to secure that the DBT treatment principles were followed in the therapies. The DBT consultation team meetings are an integrated part of the DBT-A treatment.

During covid-19 restrictions in Norway from March 2020 to March 2022 some changes in the multifamily skills training sessions were applied. For instance, shorter durations of the sessions, partial digital sessions and physical attendance, and some sessions were even cancelled. In addition, some of the individual sessions were digital or by telephone.

### Measures

Demographic information (i.e., age, sex, if their parents lived together) and the patients’ DSM-IV Axis 1 diagnosis were collected from their medical record. The DSM-IV Axis 1 diagnoses were assessed by semi-structured clinical interviews, such as Kiddie SADS [28], MiniPlus [29] and DAWBA [30] at the outpatient clinics prior to referral to DBT-A treatment. The Structured Clinical Interview for DSM-IV (SCID-II) [31] was conducted by the DBT-A therapist prior to starting DBT-A to measure traits of BPD. SCID-II has shown excellent inter-rater reliability for Axis II disorders in several studies [32, 33]. All reported measures are self-report provided by the participating adolescent.

The *primary outcomes* were as follows: self-harm episodes, hospital admissions caused by suicidal behavior, and suicide attempts. Self-reported self-harm episodes were reported weekly and organized into 5-weeks intervals before, during and after treatment. Self-reported suicide attempts prior to and during DBT-A were measured. Hospital admissions caused by suicidal behavior prior to and during DBT-A were extracted weekly from the hospital’s medical record during treatment. The primary outcomes were assessed by clinical interviews in the individual sessions, the hospital admission record and by the DBT-A diary card and registered in the patients’ medical records. For the purpose of the quality assessment register, these data are extracted from the medical record and included in the register.

*Secondary outcomes* were suicidal ideation, urge to self-harm, and self-perceived levels of sadness and happiness, and were measured by the weekly DBT-A diary cards [8]. The secondary outcomes were rated by the adolescents on a 5-point Likert scale, ranging from 0 (not at all) to 5 (extremely strong). There was no procedure to reconcile differences in ratings, as the ratings were self-reported by the patients only. However, the diary cards are continuously reviewed with the individual therapist in the individual sessions. The secondary outcomes are an integrated and standardized part of the manualized DBT-A diary cards [8], and are not administered for study purposes. These data were extracted from the DBT-A diary cards at 5-week intervals during treatment. This interval was chosen because it aligns with the time period of the 5 modes of skills the patients learn in the multifamily skills training groups.

As the quality assessment register was based on data collected as part of routine clinical practice, the inclusion of additional assessment instruments was not allowed by the hospital’s data protection officer to limit strain on the patient population. All measures used for the current study were entered in the patients’ medical record by their individual therapist at baseline, and weekly during treatment. After treatment, the medical record was reviewed, and data used for the quality assessment register of DBT-A was extracted.

### Ethics

The quality assessment register did not require consent from the patients upon establishment, as the register was intended to be used for internal evaluations of the clinical services only and no consent for this purpose was needed. The Regional Committee for Medical Research Ethics (ID469656) required consent from participants in order for their data to be used for research purposes. Informed consent was given retrospectively up to five years after the end of treatment. Adolescents aged 16 years or more provided a written consent on their own behalf. For adolescents under 16 years both parents needed to provide a written consent on their behalf. Of the 103 invited participants, 41 gave a consent. Participants received no financial compensation for their participation.

### Statistics

Statistical analyses were performed with IBM SPSS Statistics 29.0 (IBM [34]). Change in self-harm over time was analyzed using linear mixed models for repeated measures, including random effects and random intercept. Data from all participants collected at pre-treatment were included in the linear mixed model analysis, adhering to the intention-to-treat principle [35]. Missing data were estimated from the observed data based on the assumption of data missing at random, using a restricted maximum likelihood as recommended by Chakraborty and Gu [36]. Within-group effect sizes were calculated and presented as Cohen’s *d* and were based on estimated and observed changes in means from pre-treatment to the following five intervals: 1–5, 6–10, 11–15,16–20 weeks and post-treatment. The standard interpretation of Cohen’s *d* was used [37].

## Results

From January 2017 to July 2023, a total of 230 adolescents were referred to and initially screened for inclusion criteria and offered DBT-A treatment. Of these, 103 agreed to participate in DBT-A and received the treatment, whereas 41 (40%) provided a written informed consent to be included in the current study. Due to the lack of consent from the remaining 62 participants, regulations from the Regional Ethical Committee for Medical Research Ethics does not allow us to present any information about these participants. Four of the 41 adolescents that consented to participate in the study were drop-outs from the DBT-A treatment. Forty-one adolescents with a mean age of 15.8 (*SD* = 1.0, range 14 – 17), and 85.4% females, gave informed consent. The participants had received DBT-A treatment between 2017 and 2023.

**Fig. 1:**
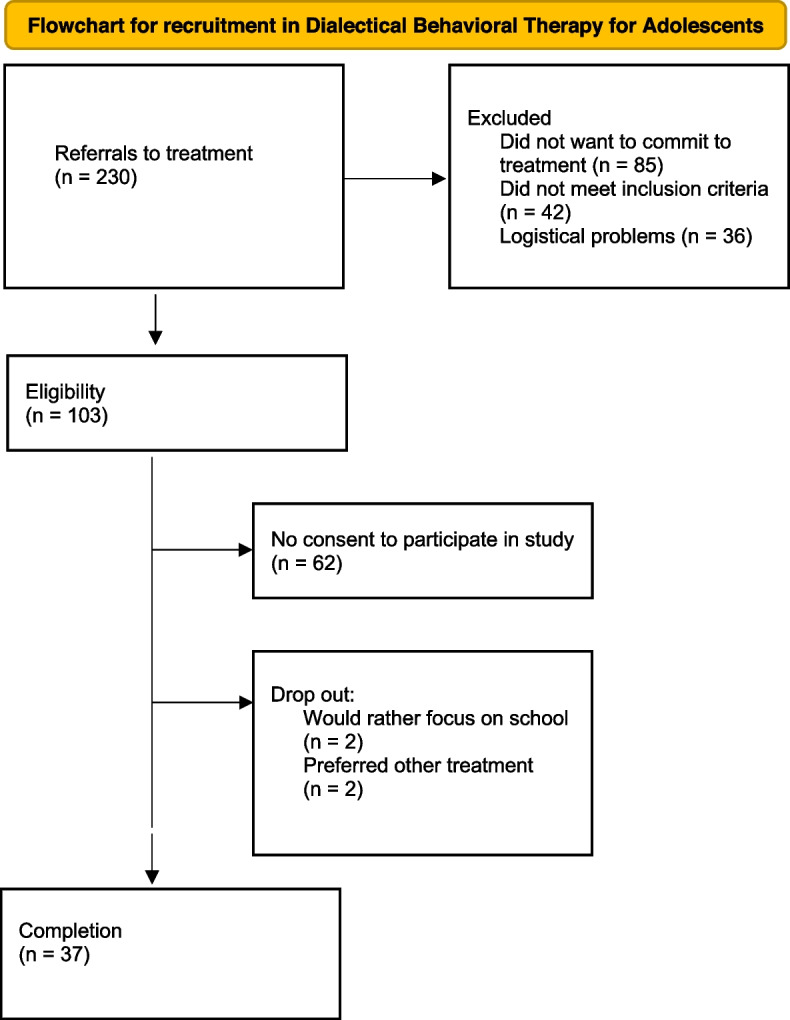
Flowchart for the recruitment process of participants in Dialectical Behavioral Therapy for adolescents in an outpatient clinic. Note. n: Number of participants; DBT-A: Dialectical Behavior therapy for Adolescents

See Fig. 1 for a flowchart of the study inclusion procedure.

### Sample characteristics

Baseline demographic characteristics, diagnostic information, pre-treatment suicide attempts, emergency hospital admissions due to self-harm, and prior use of CAMHS departments are displayed in Table 1.

**Table 1:** Demographic, diagnostic data, and earlier use of mental health services (*N*= 41)

**Variable**	**DBT-A (*n* = 41)** **N**	**%**
Female sex	35	85.4
Parents currently living together	18	43.9
Current psychopharmacotherapy	24	58.5
Current DSM-IV Axis I or Axis II diagnosis		
Any depressive disorder	16	39.0
PTSD	4	9.8
Any anxiety disorder	3	7.3
ADHD	8	19.5
BPD	2	4.9
	**Mean (SD)**	**Range**
Age (y)	15.8 (1.0)	14—17
BPD criteria fulfilled (n)	3.8 (1.9)	0—9
Current DSM-IV Axis I disorders (n)	1.5 (0.9)	0—3
Use of CAMHS departments prior to DBT-A (n)	2.1 (0.8)	1—4
Lifetime suicide attempts prior to DBT-A (n)	1.3 (1.5)	0—6
Lifetime hospital admissions prior to DBT-A (n)	1.2 (1.5)	0—6

*DBT-A *Dialectical Behavioral Therapy for Adolescents, *N* Number of participants, *DSM-IV *Diagnostic and Statistical Manual of Mental Disorders, *PTSD *Posttraumatic stress disorder, *ADHD *Attention Deficit/Hyperactivity Disorder, *BPD *Borderline personality disorder, *SD *Standard deviation *Y* Years, *N* Number, *CAMHS* Child and Adolescent Mental Health Services

### Treatment course

The patients began the 20-week DBT-A treatment after they had attended an average of 4 (*SD* = 1.6) pre-treatment sessions. The total duration of the treatment was therefore 24 weeks. In addition to the pre-treatment sessions, the patients attended an average of 17.9 (*SD* = 4.7) individual sessions, 14.7 (*SD* = 3.4) multifamily skills training group sessions and 4.6 (*SD* = 4.1) brief intersession telephone consultations. Four of the 41 patients dropped out during DBT-A treatment; two patients because they would rather focus on school and two decided during DBT-A that they preferred another therapy. Twenty-three of the participants in the study received DBT-A during covid-19 restrictions in Norway from March 2020 to March 2022. As there were some changes in the multi-family skills groups during the pandemic, we examined the potential difference in changes on self-harm between participants pre-pandemic (2017 – 2020) and during the pandemic (2020 – 2022) and the findings were not significant (*p* = 0.915).

### Primary outcomes

**Fig. 2:**
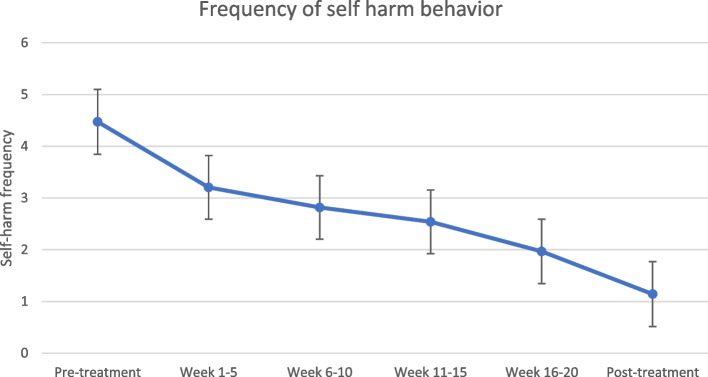
Frequency of self-harming episodes during DBT-A (*N* = 41)

The within group effect sizes for the estimated means of self-harm episodes were found to be moderate from pre-treatment to 1–5 weeks (*d* = 0.64), and large from pre-treatment to 5–10 weeks (*d* = 0.84), 11–15 weeks (*d* = 0.99), 16–20 weeks (*d* = 1.26) and post-treatment (*d* = 1.68). The self-harm episodes during the treatment course are presented in Fig. 2 and Table 2. Missing data was 23 out of 234 (9.8%) self-harm measure time points and were handled by estimation. See Table A in the appendix for the observed means. Regarding hospital admissions due to suicidal behavior, nine patients were admitted during the 20-week treatment course. Twenty-two patients had at least one admission to the hospital for suicidality prior to DBT-A (lifetime).

**Table 2:** Estimated means, standard deviations and effect sizes for the self-harm outcome measure

Assessment	*M*	*SD*	*95% CI*	*ES*^*w*^ *Pre to 1–5 weeks* *Pre to 6–10 weeks* *Pre to 11–15 weeks* *Pre to 16–20 weeks* *Pre to post-treatment*
Pre-treatment	4.47	1.98	3.85 – 5.10	
1 – 5 weeks	3.21	1.94	2.59 – 3.82	0.64
6 – 10 weeks	2.82	1.94	2.20 – 3.43	0.84
11 – 15 weeks	2.54	1.94	1.93 – 3.15	0.99
16 – 20 weeks	1.97	2.00	1.35 – 2.59	1.26
Post-treatment	1.14	1.99	0.51 – 1.77	1.68

*M* Estimated means, *SD* Standard deviations, *CI* Confidence intervals, *ES*^*w*^ Cohen’s *d* within-group effect size

Five of the nine patients that were admitted had suicide attempts during the DBT-A treatment course. There were too few incidents to statistically analyze changes in suicide attempts related to DBT-A treatment, but 24 patients of the included patients had suicide attempts prior to DBT-A. No suicides were completed during treatment.

### Secondary outcomes

The change in self-reported suicidal ideation across the treatment span showed a mean of 1.65 in week 1 to 1.33 in week 20 but was not significant. Similarly, the change in mean self-reported urge to self-harm from 1.78 in week 1 to 1.46 in week 20, was not significant. There were no significant changes in self-reported feelings of sadness and happiness across the treatment course. See Figs. 4, 5 and 6 in the appendix for the secondary outcomes.

## Discussion

The aim of this study was to examine changes in self-harm, suicide attempts and hospitalizations from suicidal behavior and self-harm among adolescents receiving a 20-weeks DBT-A program as part of routine clinical practice. We also wanted to examine whether there would be changes in suicidal ideation, self-harm urges, self-perceived sadness, and happiness from pre-, to post-treatment. In the present follow-up cohort study based on register data, adolescents receiving DBT-A reported significantly reduced self-harm frequency when DBT-A was delivered as part of routine clinical practice. The observed reduction in self-harm frequency from pre-treatment to post-treatment was large. There was a moderate reduction from pre-treatment to weeks 1–5, and the reduction in self-harm frequency increased throughout the treatment-course. No statistically significant change was found in self-reported suicidal ideation, urge to self-harm and feelings of sadness and happiness. A number of emergency hospital admissions and suicide attempts were recorded during the treatment period, but no suicides were completed.

Overall, there is a limited number of studies conducted in routine clinical practice investigating treatment for self-harm in adolescents, despite the increasing prevalence of self-harm in many countries [13]. A Cochrane review from 2021 found positive effects of DBT-A on reducing repetition of self-harm, but recommended further trials of DBT-A in different samples and settings [13]. When DBT-A was compared to treatment as usual, the Cochrane review found a reduction in self-harm post-intervention based on four RCTs with 270 participants [13]. Our study observed similar trends, with large and immediate reductions in self-harm frequency found in the participants receiving DBT-A in a routine clinical practice.

The present findings are in accordance with Kothgassner and colleagues’ systematic review and meta-analysis who found large within-group effects on self-harm in their DBT-A pre-post evaluations. The sample of patients in the current study is comparable to the inclusion criteria, age and gender in 10 of the 13 pre-post studies included in their systematic review and meta-analysis, supporting the feasibility of DBT-A on this population [12].

A large reduction in self-harm from pre- to post-treatment was found in a RCT in a CAMHS setting in Norway comparing DBT-A to treatment as usual, a clinical setting similar to the current study [38]. The results from this study also found a large reduction in the severity of suicidal ideation from baseline to post-treatment [38]. In the current cohort study, no significant reduction in suicidal ideation was found. This could be explained by the different measures used, as we only used the participants’ 1–5 score of suicidal ideation on the DBT-A diary card, and extracted this every fifth week. This measure may not accurately capture changes in suicidal ideations as it is not a validated instrument such as the Suicidal Ideation Questionnaire used in the RCT [39]. However, as the current study was based on routine clinical practice, no additional measures were included in order to avoid strain on the participants or additional work for the therapists.

The results from the current study align with findings from a similar pre-post study in an outpatient setting in Germany [40]. Buerger and colleagues had similar inclusion criteria and treatment duration and found a large reduction in self-harm episodes from baseline to post-treatment [40]. However, no measures of self-harm frequency during the treatment were included in their study, which does not provide information about when the changes occurred [40]. In the current study, a moderate to large reduction in self-harm was observed from pre-treatment to week 1–5. This is an interesting finding, as this period mainly includes the pre-treatment phase where the focus is motivation and commitment to the DBT treatment. Showing commitment to DBT treatment often includes handing in the self-harm equipment (knives, razors etc.) to the individual therapist, or applying distress tolerance skills as opposed to self-harm [4]. Future studies are recommended to examine the specific mechanisms of change involved in the reduction of self-harm during DBT-A, including the pre-treatment phase and orientation sessions.

Five of the 41 participants in the present study had suicide attempts during the DBT-A treatment. However, the study sample consists of adolescents with severe symptoms, as illustrated by 24 of the patients having at least one suicide attempt prior to starting DBT-A treatment. Few studies distinguish between self-harm with and without suicidal intent in studies on DBT-A [10]. However, a systematic review and meta-analysis by Ougrin and colleagues [41] consisting of 19 RCTs on treatments of BPD in adolescents, found that when the effects of suicide attempts and self-harm without suicidal intent were examined separately, the effect on suicide attempts was weaker than the effect of treatment for self-harm without suicidal intent. A more recent meta-analysis found no effect on suicide attempts in treatments of BPD for adolescents from pre to post treatment, including DBT-A [42]. Both these studies call for more research regarding treatment strategies for reducing the risk of suicide attempts.

There could be several reasons for the secondary outcomes being non-significant. There were no comparable data prior to DBT-A regarding suicidal ideation, urge to self-harm and feelings of sadness and happiness. The patients could have had a symptom relief during the pre-treatment phase, prior to the first measure in week one that was not captured. This could have been the case regarding the reduction in self-harm and could explain the lack of statistically significant findings for the secondary outcomes. Furthermore, COVID-19 affected the treatment from March 2020 to March 2022, as municipalities in the hospital’s catchment area had several strict lockdowns that periodically prevented individual face-to-face sessions and multifamily skills training groups. As such, the DBT-A treatment received during this period of time was sub-optimal and could have affected treatment results leading to a lack of improvement in the secondary outcomes. Additionally, several studies have demonstrated an increase in various mental health symptoms in general, and in self-harm among adolescents in particular during the COVID-19 pandemic [43, 44]. It is possible that this could have affected the adolescents in our study that received DBT-A treatment during the pandemic even though no differences in self-harm was found between participants pre- and during the pandemic.

Another consideration is that only 103 out of 188 patients referred to DBT-A treatment were found eligible for treatment, and hence, the current study. This was mainly because they did not want to commit to or consent to the treatment. A possibility is that they refused the treatment because it is time-consuming and requires a lot of motivation for change. This is in accordance with a study on drop-out from therapies by Behl and Rajagopal [45]. The authors argued that distance to therapy and time scarcity could prevent people from seeking therapy, in addition to lack of motivation, denial of having a problem or stigma related to seeking therapy.

Of 103 possible participants, only 40% provided consent to participate in the study. With 60% not providing consent, this may have caused a selection bias as participants that did not consent may share characteristics that differ from the study sample. There may be several reasons for the lack of consents. The request for consent was made retrospectively, and for some patients, almost five years after they had finished their DBT-A treatment. This group of patients may be particularly challenging to reach such a long time after treatment as they have, at least previously, had quite severe challenges. It might be that the non-consenters did not achieve any improvements, they might have had relapses or even been dissatisfied with the treatment. Ethical permission was not provided to compare those that gave consent and who did not, and it raises questions about the representativity of our sample.

It is probable that a proportion of the adolescents that declined DBT-A treatment could have received benefits from the treatment if they had committed to and accepted DBT-A. Improved knowledge regarding the reasons for declining DBT-A treatment, as well as drop-out during treatment are important areas of research for future studies.

### Strengths and limitations

A strength of the current study is the routine clinical care setting and the use of data based on a systematic quality assessment register. This leads to the study population being clinically representative of DBT-patients in outpatient settings and hence ensuring a high generalizability and external validity of the findings [46]. In addition, only four of the participants from the study sample dropped out from treatment.

Our results should be viewed in the context of some limitations. The study is based on a quality assessment register. The lack of a control group or an alternative treatment group to compare whether the changes were due to unspecific therapeutic effects or natural fluctuations limits the nature of the conclusions that can be drawn. Although this practice is representative of clinical care provided outside of research trial settings, it makes it difficult to draw firm conclusions regarding the effect of DBT-A. Participants may have accepted to participate in DBT-A at a particularly difficult point in time, and improvement may have represented regression to the mean. Further, since our study did not include a follow up, we do not know the long-term stability of treatment success over time. Future studies should utilize study designs that allows for inference of causality by including a control group and long-term follow ups.

An additional limitation is that our primary and secondary outcome data were extracted from medical records and DBT-A diary cards, and except from the number of hospital admissions caused by suicidal behavior, data were based on self-report. Self-report measures have a potential influence of demand characteristics. Additionally, the lack of validated outcome measures should be considered when interpreting the findings of the current study, and future studies should seek to implement standardized assessment tools in DBT-A treatment.

Another limitation is that no standardized adherence measures were included in the study to ensure treatment adherence among the DBT-therapists as it was conducted in a naturalistic setting. However, there are several integrated aspects in DBT-A that aim to ensure treatment adherence, such as the weekly consultation team meeting, rigorous training and supervision, and multiple therapists being involved in each patient’s treatment [33].

Finally, a considerable proportion of the participants were in pharmacological treatment in the pre-treatment phase, but we did not assess use of medication and change throughout the DBT-A treatment. The pharmacological treatment were mainly antidepressants and ADHD medication. It is possible that the use of medications could have influenced the reduction found in self-harm episodes. Considering these limitations, the findings of the current study may provide useful information on the evaluation of DBT-A in routine clinical care and may further inform future efforts to improve outpatient DBT-A for self-harm and suicidal ideation in adolescents.

## Conclusion

We examined outcomes related to suicidality in adolescents receiving DBT-A, delivered as standard care in a public mental health service, in a follow-up cohort study based on data from a quality-assessment register. The findings showed large reductions in self-harm episodes in adolescents receiving DBT-A treatment. The observed reduction in self-harm was immediate and improved throughout the entire treatment course. Future studies with more psychometrically sound measures, independent raters and long-term follow-up assessments should seek to examine the effect of the specific modalities on self-harm in DBT-A treatment. In addition, further investigations of the reasons for drop-out and lack of commitment to DBT-A are warranted, as little is known about these patients and their prognoses.

### Supplementary Information

Supplementary Material 1.

## Data Availability

The data that support the findings of this study are not openly available due to reasons of sensitivity and are available from the corresponding author upon reasonable request. Data are located in controlled access data storage at Helse-Bergen.

## Abbreviations

BPD: Borderline personality disorder
DBT: Dialectical behavior therapy
MBT: Mentalization-based therapy
DBT-A: Dialectical behaviour therapy for adolescents
SD: Standard deviation
ES: Effect size
d: Cohen’s *d*
RCT: Randomized-controlled trial
CAMHS: Child and Adolescent Mental Health Services
SCID-II: The Structured Clinical Interview for DSM-IV version 2
