# Supplementary material for: Evaluation of dialectical behavior therapy for adolescents in routine clinical practice: a pre-post study
Source: BMC Psychiatry. 2024 Jun 14;24:447. doi: 10.1186/s12888-024-05876-z (PMC11177375; doi:10.1186/s12888-024-05876-z)

Table A

Observed means, Standard Deviations and Effect sizes for Self-harm outcome measure

Assessment	*n*	*M*	*SD*	*ES^w^*<br>*Pre to 1-5 weeks*<br>*Pre to 6-10 weeks*<br>*Pre to 11-15 weeks*<br>*Pre to 16-20 weeks*<br>*Pre to post-treatment*
Pre-treatment	34	4.56	2.26	
1-5 weeks	36	3.17	2.17	0.63
6 – 10 weeks	36	2.78	2.29	0.78
11 - 15 weeks	36	2.50	1.73	1.02
16 – 20 weeks	35	1.91	1.50	1.38
Post-treatment	34	1.12	0.69	2.06

Note.; n: number of participants; M: estimated means; SD: standard deviations; ES^w: Cohen's *d* within-group effect size.

Figure 4: Mean intensity of suicidal ideation

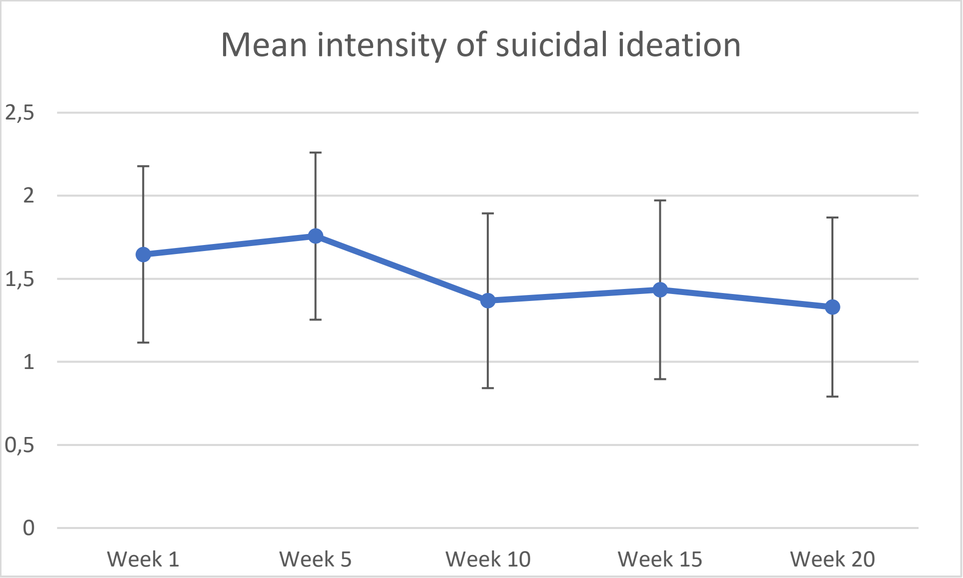

Figure 5: Mean intensity of urge to self-harm

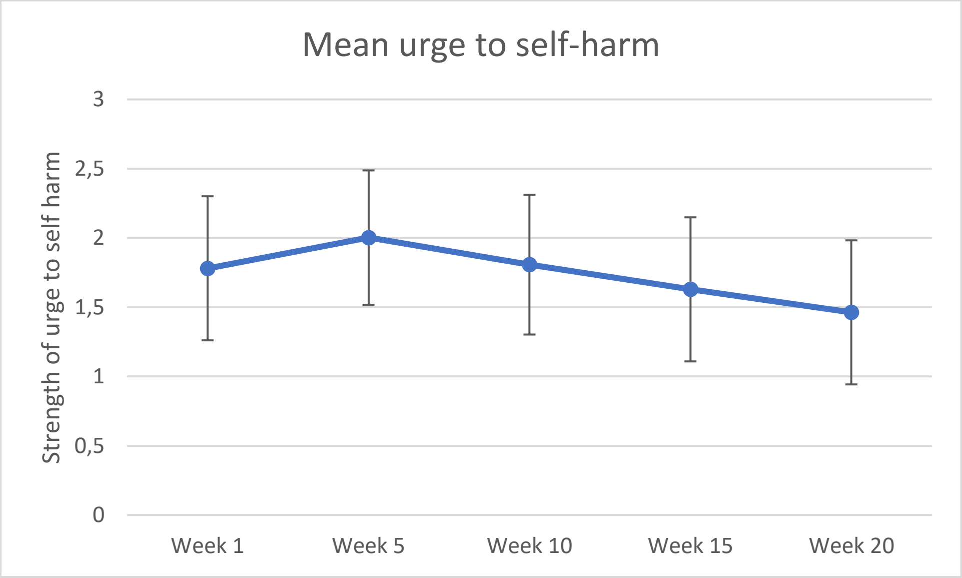

Figure 6: Feelings of sadness and happiness

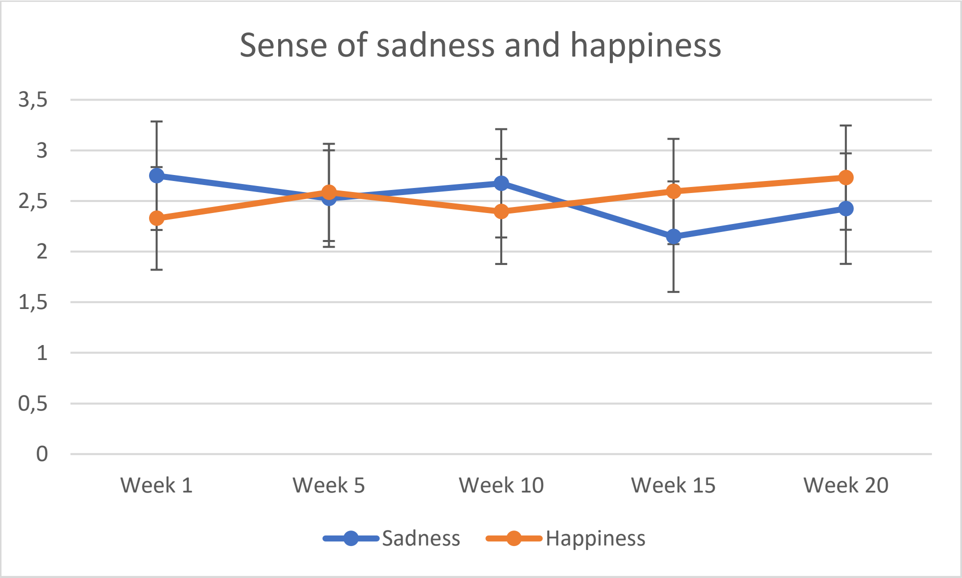

Supplement: Supplementary file 1 — Supplementary Material 1. [file 12888_2024_5876_MOESM1_ESM.pdf]
